# Artificial Intelligence–Based Consumer Health Informatics Application: Scoping Review

**DOI:** 10.2196/47260

**Published:** 2023-08-30

**Authors:** Onur Asan, Euiji Choi, Xiaomei Wang

**Affiliations:** 1 School of Systems and Enterprises Stevens Institute of Technology Hoboken, NJ United States; 2 Department of Computer Science Stevens Institute of Technology Hoboken, NJ United States; 3 Department of Industrial Engieering University of Louisville Louisville, KY United States

**Keywords:** consumer informatics, artificial intelligence, mobile health, mHealth, patient outcomes, personalized health care, machine learning, digital health, mobile phone

## Abstract

**Background:**

There is no doubt that the recent surge in artificial intelligence (AI) research will change the trajectory of next-generation health care, making it more approachable and accessible to patients. Therefore, it is critical to research patient perceptions and outcomes because this trend will allow patients to be the primary consumers of health technology and decision makers for their own health.

**Objective:**

This study aimed to review and analyze papers on AI-based consumer health informatics (CHI) for successful future patient-centered care.

**Methods:**

We searched for all peer-reviewed papers in PubMed published in English before July 2022. Research on an AI-based CHI tool or system that reports patient outcomes or perceptions was identified for the scoping review.

**Results:**

We identified 20 papers that met our inclusion criteria. The eligible studies were summarized and discussed with respect to the role of the AI-based CHI system, patient outcomes, and patient perceptions. The AI-based CHI systems identified included systems in mobile health (13/20, 65%), robotics (5/20, 25%), and telemedicine (2/20, 10%). All the systems aimed to provide patients with personalized health care. Patient outcomes and perceptions across various clinical disciplines were discussed, demonstrating the potential of an AI-based CHI system to benefit patients.

**Conclusions:**

This scoping review showed the trend in AI-based CHI systems and their impact on patient outcomes as well as patients’ perceptions of these systems. Future studies should also explore how clinicians and health care professionals perceive these consumer-based systems and integrate them into the overall workflow.

## Introduction

### Background

Health information technologies have been a fundamental part of health care delivery systems in the last decade, advancing the care significantly [1]. Traditionally, health information technologies are classified into 3 categories, namely clinical technologies (eg, electronic health records and clinical decision support systems), collaborative technologies (eg, patient portals), and consumer technologies (eg, mobile health [mHealth] app) [2]. As health care shifts to more community and public settings to enable preventive and patient-centered care, patients have increasingly engaged with self-care management using various consumer health technologies [3,4]. The use of various consumer health informatics (CHI) tools has increased dramatically during the recent pandemic [5-7]. CHI is a health information technology that uses data collected by any electronic tools, technologies, or applications to interact directly with consumers to provide individualized information and assist consumers or patients in better self-managing their health [8]. Furthermore, the potential benefits of health information technologies have significantly improved with the integration of novel artificial intelligence (AI) algorithms into both clinical and consumer settings [9]. The exponential growth of AI-based applications also results in a significant shift in the field of health care [10,11] and opens a new age of AI health care [9]. According to Fortune Business Insights, the potential investment for AI-based applications in the global health care market will reach US $164.10 billion by 2029 compared with the investment of US $13.82 billion made in 2022 [12].

The increased adoption of AI systems in the health care field has also contributed to increasing significance of and attention toward AI systems in health care domain research. Over the years, researchers have extensively studied incorporating AI into clinical health IT applications [13] such as patient outcome prediction [14,15] or improving the accuracy of clinical AI systems [16-18]. However, the use of AI in health care is not limited to clinical health IT applications. AI systems also have the potential to improve the patient-centeredness of health care [19] by enhancing patient health self-management tools such as smartphone apps [20,21], assistant robots [22,23], and self-monitoring devices [24-26], which are various types of CHI tools. It is essential to understand how patients and consumers perceive and experience the use of these technologies.

### Objective

Given the potential growth and benefits of AI-based CHI systems, it is important to understand how these systems would benefit health care stakeholders and academia to steer the future of AI development toward successful patient self-care management. In the past, a number of literature reviews have discussed how patients generally view clinical AIs that are implemented in clinic and hospital settings and used by health care providers; however, there is a lack of literature on the outcomes and patients’ perceptions of AI-based CHI systems. In this scoping review, we focused on (1) how AI-based CHI systems impact patient outcomes and (2) how patients perceive AI-based CHI systems. We also discussed the types of AI models used in the identified CHI systems. To the best of our knowledge, this is the first review to analyze patient perceptions of and outcomes after using AI-based CHI systems [27-31].

## Methods

### Protocol Registration and Information Sources

We conducted a scoping review to explore the trending literature on consumer-based AI systems in health care. Scoping reviews are an effective and useful strategy for synthesizing emerging concepts and topics in a specific domain [32]. This scoping review is reported according to the PRISMA-ScR (Preferred Reporting Items for Systematic Reviews and Meta-Analyses extension for Scoping Reviews) guidelines (Multimedia Appendix 1) [32]. Our protocol was registered with the Open Science Framework on June 14, 2022. We searched all peer-reviewed publications in the PubMed database published before July 2022 to identify studies that were within this review’s scope and met the eligibility criteria.

### Search Strategy

**Figure 1:**
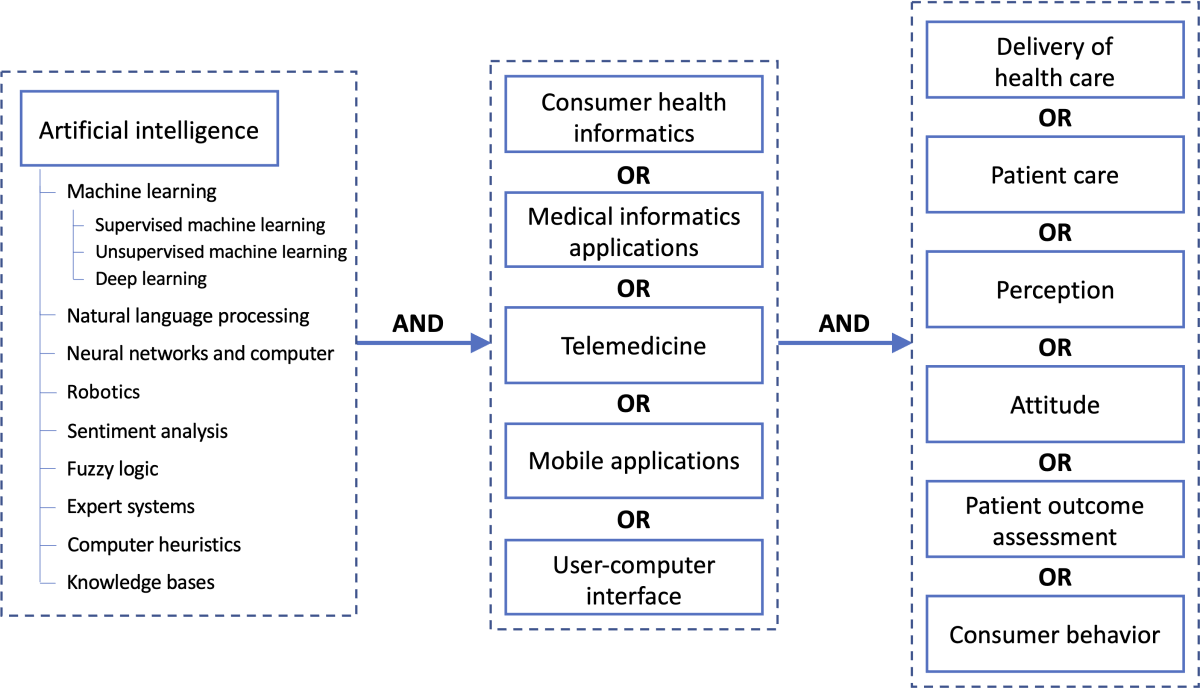
Conceptual framework of the Medical Subject Headings terms used in the literature review search.

We followed a systematic method for creating search terms to capture all related and eligible papers in the searched database. Keywords used for the literature search were selected through a preliminary literature review and then adjusted based on the feedback from content experts and our institution’s librarian. We specifically had a few meetings with the librarian, during which we refined the search strategy to ensure that all papers related to AI-based CHI were considered in our review and determined the Medical Subject Headings (MeSH) terms. MeSH terms were used as search keywords to optimize the search strategy [33]. MeSH terms are composed in a hierarchical structure, often referred to as a MeSH tree, branching from general to detailed terms. By searching a MeSH term, its synonyms, branch terms, and their synonyms are automatically included in the search query. For example, synonyms of “artificial intelligence” (MeSH), such as “machine intelligence,” “AI,” and “computer intelligence,” are searched simultaneously, and all the child MeSH terms, such as “machine learning” (MeSH), “natural language processing” (MeSH), and “neural networks” (MeSH), are concurrently included. The hierarchy of “artificial intelligence” (MeSH) is depicted in Figure 1, and its synonyms are listed in Textbox 1. In this study, we grouped the appropriate parent MeSH terms and used Boolean (AND and OR) operators to identify all relevant studies that matched our scope and inclusion criteria. Figure 1 illustrates all the combinations of MeSH terms used in the search (eg, “Artificial Intelligence” [MeSH] AND “Consumer Health Informatics OR Mobile Applications” [MeSH] AND “Patient Care” [MeSH]).

**An example of Medical Subject Headings (MeSH) term synonyms.**

Machine intelligence

- Intelligence and artificial
- Intelligence and machine
- Knowledge acquisition (computer)
- Vision system and computer
- Representation and knowledge (computer)
- Acquisition and knowledge (computer)

Artificial intelligence

- Intelligence and computational
- Computer reasoning
- Vision systems and computer
- Computer vision systems
- Knowledge representation (computer)

Computational intelligence

- Reasoning and computer
- Systems and computer vision
- Computer vision system
- System and computer vision
- Knowledge representations (computer)

### Definitions of AI and CHI

AI is a field in computer science that aims to recreate human intelligence using computer systems [34]. It can also be defined as a computer program that can make intelligent decisions [35]. It is an umbrella term that encompasses many subfields, including, but not limited to, machine learning (ML), robotics, computer vision, and expert systems. In this review, we incorporated all terms under the MeSH term “artificial intelligence” (MeSH), including “machine learning” (MeSH) and “natural language processing” (MeSH). The synonyms of AI are listed in Textbox 1.

According to Eysenbach [36], the general definition of CHI is as follows: a branch of medical informatics that analyzes consumers’ need for information, studies and implements methods of informing consumers, models consumers’ preferences, and integrates them into medical information systems. For the scope of our review, we have incorporated the definition of CHI application by Gibbons et al [37]. The authors have defined CHI applications as any electronic tool, technology, or system that (1) is primarily designed to interact with health information users or consumers, (2) directly interacts with the consumer who provides personal health information to the CHI system, and receives personalized health information, and (3) the benefits provided to the consumer may be accessed with the help of a health care professional but is not dependent on a health care professional [37].

In this paper, we focused on reviewing AI-based CHI systems, which are defined as AI systems that interact directly with patients or consumers to encourage and benefit the management of their health with or without a health care professional.

### Inclusion and Exclusion Criteria

This review included peer-reviewed studies that satisfied two primary conditions:

1. The study needed to focus on an AI-based CHI tool or system. Articles that do not focus on AI-based CHI tool or system were excluded from the review. Therefore, any AI-based CHI that involves a patient-facing system, with or without the presence of a health care professional, was considered.
2. The study needed to report the impact of AI-based CHI on health-related outcomes or patients’ perceptions of AI-based CHI.

We excluded studies if any of the following conditions were met:

1. Patient was not the primary consumer or user.
2. The study focused only on the accuracy of the AI system and did not collect any data from patients or consumers.
3. The study focused on medical chatbots. We excluded chatbots from our review because there has been extensive research and literature reviews specifically in this area [38-40].
4. The study involved secondary research, such as a review, commentary, or conceptual paper.
5. The study was not published in English.

### Study Selection and Data Synthesis

**Figure 2:**
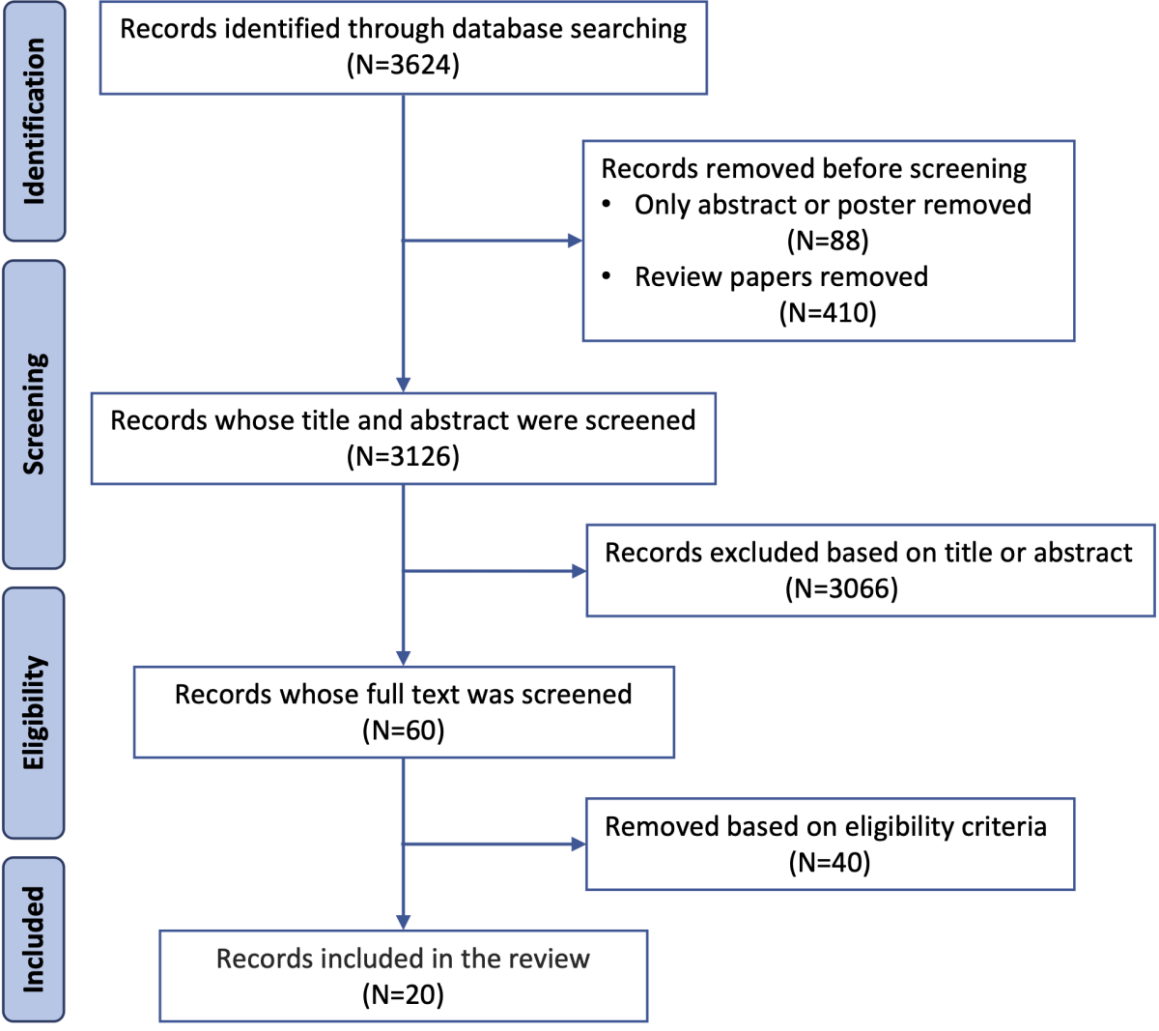
The flowchart for searching and selection process.

The authors EC and OA together assessed the collected studies for eligibility. We first removed any duplicates and screened the studies by reviewing the titles and abstracts. We created an Excel (Microsoft Corp) spreadsheet to take detailed notes on each study. This sheet included information regarding the objective, AI-based CHI system, reported outcomes, AI algorithm, performance of the AI algorithm, type of users, sample size, and additional notes. The 2 authors (OA and EC) independently took notes on each study, after which a detailed comparison was made between their respective notes. To minimize selection bias, all discrepancies were resolved through discussion, requiring consensus from both the reviewers. A data abstraction form was used to record standardized information from each paper. We finalized the selection of studies to be included in the review by following this process. The study selection details are shown in Figure 2.

## Results

### Study Selection

Figure 2 presents the flowchart of the selection process for this scoping review. The initial search conducted on PubMed using a set of queries returned 3624 records. We used EndNote (version 20.3; Clarivate) to manage the filtering and removal processes. First, we removed all review, opinion, and perspective papers (410/3624, 11.31%) and posters or short abstracts (88/3624, 2.43%). The authors (EC and OA) then performed a second round of filtering by reading the abstracts and titles of the remaining papers (3126/3624, 86.26%). The screening process was carried out in accordance with the inclusion and exclusion criteria detailed in the *Inclusion and Exclusion Criteria* section, resulting in the selection of 1.92% (60/3126) of papers for a full-text review. The authors then removed 67% (40/60) of papers based on the full-text review. Therefore, with consensus from both the authors, a total of 50% (20/40) of studies were included in this review.

Table 1 summarizes the design of the selected reports (n=20), including the objective of the study, the application field of the AI-based CHI system, and the patient perceptions of or outcomes after using the system. The total number of the papers shows that there is still immaturity in this area and that there is a need for further work given the technological advancement. However, half (10/20, 50%) of the papers were published within the last 2 to 3 years, which shows a recent growth in this domain of the research.

**Table 1:** The study design and reported findings of final selected papers.

Study, year, place of publication	Objective	AI^a^-based CHI^b^ system		Patient sample size, n	Patient outcomes and perceptions
		Type	Name		
Mihailidis et al [41], 2008, Canada	To assist people with dementia in performing activities of daily living	Assisted living device	COACH^c^	6	Patients showed improvement in handwashing by using COACH and completed more steps independently without caregiver’s assistant. Higher FAS^d^ score was observed.
Curran et al [42], 2010, the United Kingdom	To propose a mobile app that provides personalized insulin injection amount for patients with diabetes	mHealth^e^	INNSULIN^f^	6	Patients gave very favorable feedback regarding the system’s trustworthiness, as the system involved clinician monitoring.
Guidali et al [43], 2011, Switzerland	To develop a robot-supported rehabilitation system for activities of daily living	Rehabilitation device	ARMin III	10	Patient’s motivation could be rated on a scale of 1-5, and all the patients rated 5. They reported that they were very eager to train. The mean total execution time for the task was approximately 4 times higher in patients than in healthy participants.
Leuty et al [44], 2013, Canada	To enable more independent access to art creation for the well-being of older adults with dementia	Assisting device for older adults with dementia	ePAD^g^	6	4 (67%) out of 6 patients expressed excitement about the novelty of the device and ease of features and were satisfied with the art they created. Patients felt confused and frustrated, as the device had a gap between the actual brush and computer screen. Patients indicated that the given prompts were not attention grabbing. Therefore, they did not respond in the intended fashion.
van der Heijden et al [45], 2013, the Netherlands	To predict and detect exacerbations and help patients self-manage their disease to prevent hospitalization	mHealth	Aerial	5	Patients indicated that they would be willing to use such a system in a home-care setting. Patients found the system to be useful for gaining insights into the disease, to be easy to use, and to not be intrusive.
Robles-Bykbaev et al [46], 2015, Ecuador	To develop an expert system to aid speech and language pathologists in the generation and updating of therapy plans	mHealth	SPELTA^h^	53	Patients (children) showed high levels of motivation during the speech and language therapy activities.
Hezarjaribi et al [47], 2018, the United States	To propose a voice-based mobile nutrition monitoring system to self-manage diet and health	mHealth	Speech2Health	10	Patients rated the system 3.89 out of 5 on user acceptance. User satisfaction, which indicates the ease of use, was rated 4.25 out of 5. However, patients indicated that other methods such as text provided more privacy than voice- or image-based systems. Patients rated the system 3.25 out of 5 on likelihood of use.
Forman et al [48], 2019, the United States	To predict dietary lapses and deliver a targeted intervention designed to prevent the lapses from occurring	mHealth	OnTrack	44	Participants rated the app as moderately useful and enjoyable and indicated a somewhat positive behavioral intention to use the app. They reported that the interventions were moderately helpful. They demonstrated an average of 3.13% weight loss. Overall, 43% (19/44) of the participants achieved a weight loss of at least 3%, and 36% (16/44) of the participants achieved a weight loss of ≥5%. Participants generally perceived most risk alerts to be accurate and appropriate.
Poirier et al [49], 2019, Canada	To develop an intuitive control interface system based on voice commands for current and potential assistive robot users	Assisted living device	NR^i^	5	Participants did not express that vocal control interface improved their performances. However, they found the vocal control interface useful, easy to learn, easy to use, less cumbersome, as requiring less concentration, and as requiring less effort and to be more intuitive than joysticks.
Ramkumar et al [50], 2019, the United States	To develop remote patient monitoring system for patients after total knee arthroplasty	mHealth	TKR	25	A total of 22 patients were available for follow-up interviews, where all patients expressed that they found the remote patient monitoring system engaging and that they would recommend the system to other patients. The difficulty of the technology use was rated fairly low, 2.6 out of 10. However, 36% (8/22) of the patients commented that the low battery life of the system needs improvement. The mean mobility returned to baseline within 6 weeks and exceeded preoperative baseline by 30% at 3 months.
Pelle et al [51], 2020, the Netherlands	To evaluate the short-term effects of the dr. Bart app in comparison with those of usual care on the number of health care consultations and clinical outcomes of patients with knee and hip osteoarthritis	mHealth	dr. Bart	427	No difference between the intervention (dr. Bart app) group and control (usual care) group in the number of secondary health care consultations was found. Small positive treatment effects on symptoms, pain, and activities of daily living were observed.
Meeuws et al [52], 2020, Belgium	To autonomize CI^j^ fitting by combining patient’s psychoacoustic self-testing and AI interpretation	Telemedicine	NR	6	4 (67%) out of 6 patients did not have any problem with self-testing. 4 (80%) out of 5 patients showed improvement in speech perception, with an average increase in phoneme scores of 6%. Although patients preferred the presence of a CI audiologist while fitting, 83% (5/6) of the patients expressed that they would not hesitate to do self-tests at home with remote supervision.
Carrasco-Hernandez et al [53], 2020, Spain	To provide AI-generated and tailored smoking cessation support messages	mHealth	So-Lo-Mo^k^	120	Abstinence was 2.75 times higher for app users who also received psychopharmacological treatment compared with patients who received psychopharmacological treatment alone. Patients rated the app an average of 3.97 on trust and 4.35 on satisfaction.
Burdea et al [54], 2021, the United States	To enhance poststroke rehabilitation by developing a game controller adaptable to people with disabilities	Rehabilitation device	BBG^l^	2	For all but 1 game, participant 1’s average rating/game was ≥4 out of 5. Participant 2 rated all games ≥4.7 out of 5 on the custom evaluation form. Although the participants gave feedback on adjusting the games for effective engagement, both participants strongly agreed that the system was easy to use, user-friendly, and fun to use, and they were able to learn to use it quickly.
Jayakumar et al [55], 2021, the United States	To evaluate a decision aid software that delivers education, an interactive preferences assessment, and personalized outcome estimations for patients with knee osteoarthritis	Telemedicine	NR	129	The intervention group showed better decisional quality, collaborative decision-making and satisfaction and improved functional outcomes at a follow-up appointment after using the system for 4-6 months.
Liu et al [56], 2021, the United States	To monitor and support HIV PrEP^m^ use	mHealth	aDOT^n^	20	High acceptance was observed among patients, with median System Usability Scale scores in the excellent range (80/100, 80%). App use was high, with 85% (17/20) of the participants reporting that the app helped with taking PrEP.
Frías et al [57], 2021, Spain	To develop a psychotherapeutic app for self-managing crises in BPD^o^	mHealth	B·RIGHT	25	Patients with BPD considered the app user-friendly (mean total score 4.03) and highly satisfactory (mean total score 4.02), resulting in a positive user experience (mean total score 4.09).
Santala et al [58], 2021, Finland	To detect arrhythmia using a self-monitoring app	mHealth	NR	159	User experience with the heart belt was found to be significantly better than that with the Holter ECG^p^ in a subgroup of older patients (aged >65 years). A higher proportion (123/159, 77.4% vs 112/159, 70.4%) of older patients preferred the heart belt to the Holter device (gold standard).
Park et al [59], 2022, Republic of Korea	To provide accurate exercise guides to users	mHealth	LikeFit	176	Reduction in lower back pain scores and improvement of quality of life in the MDMECA^q^ group may have resulted from a higher exercise performance rate due to correct posture advised by machine learning system. Intention to use and recommend in the MDMECA group were 4.23 and 4.49 out of 10, respectively, and were higher than those in the control group.
Shah et al [60], 2022, India	To identify normal vs abnormal retina	mHealth	Netra.AI	104	90.4% of the patients were willing to participate in an AI‐based fundus screening; 96.2% were satisfied with AI‐based screening.

^a^AI: artificial intelligence.

^b^CHI: consumer health informatics.

^c^COACH: Cognitive Orthosis for Assisting Activities in the Home.

^d^FAS: functional assessment scale.

^e^mHealth: mobile health.

^f^INNSULIN: Intelligent Neural Network for Suggesting Unambiguous Levels of Insulin via Needle.

^g^ePAD: Engaging Platform for Art Development.

^h^SPELTA: Speech and Language Therapy Assistant.

^i^NR: not reported.

^j^CI: cochlear implant.

^k^So-Lo-Mo: Social-Local-Mobile.

^l^BBG: BrightBrainer Grasp.

^m^PrEP: pre-exposure prophylaxis.

^n^aDOT: automated directly observed therapy.

^o^BPD: borderline personality disorder.

^p^ECG: electrocardiogram.

^q^MDMECA: motion-detecting mobile exercise coaching application.

As described in Table 1, most of the studies reported favorable patient perception of the AI-based CHI systems. Moreover, many studies observed improved patient outcomes through the intervention of the systems, allowing patients to better self-manage and improve their health using AI-based CHI systems. For instance, AI helped predict dietary lapses and conveniently monitor the nutrition system through speech recognition [47]. AI also provided a personalized tool for assisting patients with performing daily activities [43], supplying a wider range of options for patients with everyday needs. In addition, AI enabled an efficient self-diagnosis system on a clinical level. Table 2 outlines the AI methods used in and performance measures of the CHI system adopted by the selected studies, if reported (with “NR” denoting not reported).

**Table 2:** Artificial intelligence (AI) methods used in and performance measures of AI-based consumer health informatics systems.

Study, year	AI method or algorithms	AI performance
Mihailidis et al [41], 2008	Computer vision, flocking, and partially observable Markov decision process	NR^a^
Curran et al [42], 2010	Artificial neural network	NR
Guidali et al [43], 2011	Computer vision and nearest neighbor	NR
Leuty et al [44], 2013	NR	NR
van der Heijden et al [45], 2013	Bayesian network model	AUC^b^ of the Bayesian network model was 0.93 and that of the expert model was 0.97
Robles-Bykbaev et al [46], 2015	PAM^c^ and kNN^d^	Accuracy of 90%
Hezarjaribi et al [47], 2018	NLP^e^ and speech recognition (NLP supervised learning)	Accuracy of 97.69%
Forman et al [48], 2019	Ensemble methods (logit boost, bagging random subspace, random forest, and Bayes net)	Accuracy of 72%
Poirier et al [49], 2019	GMM^f^ for training and SVM^g^ (word or noise)	NR
Ramkumar et al [50], 2019	NR	NR
Pelle et al [51], 2020	NR	NR
Meeuws et al [52], 2020	Bayesian networks, influence diagrams, probabilistic models, and ML^h^	NR
Carrasco-Hernandez et al [53], 2020	Just HRS^i^	NR
Burdea et al [54], 2021	An adaptive algorithm for mapping baseline-detected workspace and ensuring maximum patient engagement by setting game difficulty	NR
Jayakumar et al [55], 2021	ML (not specific)	NR
Liu et al [56], 2021	Deep learning and computer vision	NR
Frías et al [57], 2021	NR	NR
Santala et al [58], 2021	Commercial AI arrhythmia analysis software (Awario, Heart2Save)	Accuracy of 97.5%
Park et al [59], 2022	Deep learning	NR
Shah et al [60], 2022	Deep learning	Accuracy of 96.5%

^a^NR: not reported.

^b^AUC: area under the receiver operating characteristic curve.

^c^PAM: partition around medoids.

^d^kNN: k-nearest neighbors.

^e^NLP: natural language processing.

^f^GMM: Gaussian mixture model.

^g^SVM: support vector machine.

^h^ML: machine learning.

^i^HRS: health recommender system.

### AI-Based CHI Systems

**Figure 3:**
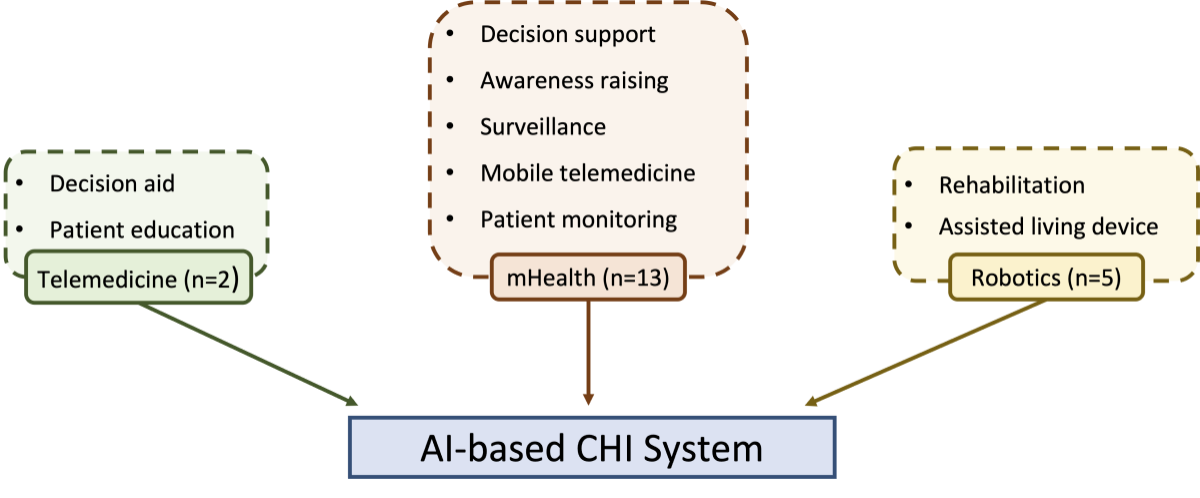
Type of artificial intelligence (AI)-based consumer health informatics (CHI) systems. mHealth: mobile health.

Various types of AI-based CHI systems were observed in this review. Figure 3 depicts the types of system in the reviewed studies as well as the observed subcategories and functions of each system type. Of the 20 papers reviewed, 13 (65%) were focused on mHealth-based systems, 5 (25%) were focused on robotics-based systems, and 2 (10%) were focused on telemedicine-based systems. The first study on AI-based CHI systems with patients focused on a robotics-based system introduced in 2008 for helping patients with dementia achieve greater independence without the assistance of caregivers [41]. The authors developed an assisted living device using computer vision to help patients with handwashing procedures. It has been discovered that the early AI-based CHI systems focused on rehabilitation systems and assisted daily living devices [43,44] following the initial publication which focused on assisting dementia patients with daily activities [41]. This introduction of an AI-based CHI system stands to reason because although AI in health care can be divided into 2 subtypes, virtual and physical [61], AI is generally acknowledged to have begun in the physical form with the invention of robots [62].

However, in recent years, the acceleration in the development of ML algorithms [63] has led to most studies being focused on the virtual realm of AI, particularly on mHealth (13/20, 65%). Notably, the first mHealth paper was published in 2010, and 10 (77%) out of the 13 included studies on mHealth were conducted within the last 5 years. Although the recent surge in AI and AI-based mobile apps anticipate a significant number of mHealth apps in AI-based CHI systems, the fact that mobile apps directly connect consumers and technology further supports the high amount of patient data obtained. Studies on rehabilitation or assisting devices were also notable as patient feedback and outcomes play a determinative role in successful device development.

Moreover, the selected AI-based CHI systems were observed in many clinical fields. Except for the studies on the rehabilitation of patients with dementia and patients with stroke (n=5), the remaining studies were dispersed across a broad range of fields. Studies were focused on general patient health systems (7/20, 35%; systems for nonchronic conditions), including systems for dietary support (2/7, 29%), drug adherence (1/7, 14%), smoking cessation (1/7, 14%), exercise support (1/7, 14%), emotional crisis management (1/7, 14%), and language therapy (1/7, 14%). Many systems in general patient care aimed to monitor patients and support self-health management, such as medication adherence [56] and monitoring nutrition and calorie intake [47]. Furthermore, some studies aimed to raise awareness and provide additional support for patients by sending personalized cessation support messages [53], providing accessible behavioral therapy [57], and offering advice on patients’ exercise postures [59].

In addition to the general health systems, chronic condition–related systems (8/20, 40%) were studied, namely those for knee osteoarthritis (3/8, 38%), diabetes (1/8, 13%), cochlear implant (1/8, 13%), arrhythmia detection (1/8, 13%), diabetic retinopathy (1/8, 13%), and chronic obstructive pulmonary disease (1/8, 13%). The goals of these systems were also similar to those of general health systems; for example, the systems for knee osteoarthritis included a patient monitoring system [50] and a system raising health awareness by suggesting small exercise goals [51]. However, there was an emphasis on decision aid and surveillance, particularly among systems related to chronic conditions. An AI decision aid providing patient education for knee osteoarthritis [55] and a decision aid for potential diabetic retinopathy [60] were observed. We also observed examples of surveillance systems, arrhythmia detection systems [58], and chronic obstructive pulmonary disease exacerbation detection systems [45]. Consequently, patient-related data were obtained in various clinical disciplines and for various objectives.

In addition to the systems’ wide range of clinical applications, the first authors’ locations were also observed to be widespread: the United States (6/20, 30%), Canada (3/20, 15%), the United Kingdom (1/20, 5%), Spain (2/20, 10%), the Netherlands (2/20, 10%), Ecuador (1/20, 5%), the Republic of Korea (1/20, 5%), Finland (1/20, 5%), Belgium (1/20, 5%), Switzerland (1/20, 5%), and India (1/20, 5%). The first authors were primarily affiliated with universities and research institutions (13/20, 65%), but some were also affiliated with hospital centers (6/20, 30%) and private businesses (1/20, 5%). This implies that the study of AI-based CHI systems for patient-centered care has attracted researchers globally, both academically and in the health care business.

### Patient Outcomes and Perceptions of AI-Based CHI Systems

Among the reviewed studies (n=20) on AI-based CHI systems that reported patient data, patient perception was reported in most of the studies (18/20, 90%). Patient perception was represented by, but was not limited to, patient satisfaction, user experience, preference, trust, intention to use, ease of use, and motivation. Several studies measured patient perception quantitatively through ratings and surveys, such as using the Technology Acceptance Model scale [48], Systems Usability Scale [56], or the 5-point Likert scale [44,47-49,53,54,60]. By contrast, some studies (2/20, 10%) collected patient perception data through qualitative methods such as interviews or verbal feedbacks [45,56].

Most of the studies reported favorable patient perceptions toward the AI-based CHI system. Many participants expressed their motivation and eagerness to use the system. A study showed that a highly satisfactory and user-friendly experience led to a positive user experience [57]. Moreover, a few studies (2/20, 10%) reported trust along with satisfaction [53,60]. Satisfaction generally gained a higher score than trust; for instance, patients rated an average of 3.97 for trust and 4.34 for satisfaction [53]. Another study reported that 71.1% of the patients were either highly satisfied or satisfied, but only 37.5% of the patients agreed that the system could replace clinicians [60]. Nevertheless, these studies indicated patients’ positive intention to use and recommend the system. Few study participants expressed confusion or frustration toward the system [44] or suggested improvements in the system, such as the improvement of the low battery life of the device [50].

In addition, patient outcomes were reported in many studies (5/20, 25%) included in this review. Patients exhibited improvements, including in handwashing performance [41], the prevention of dietary lapses [48], speech perception [52], and smoking abstinence [53]. One study reported that the decision aid system enhanced the decisional quality of patients [55]. However, a few studies (2/20, 10%) have shown that not every AI-based CHI system improved patient outcome. For example, participants in 1 study did not express that the vocal control interface improved their performance despite having positive perception toward the system [49]. Another study reported that the implementation of the system showed small positive treatment effects on patients but did not make a difference in the number of secondary health care consultation [51].

The sample size of the studies is listed in Table 1. The sample size ranged from 5 to 427 patients. Approximately half (9/20, 45%) of the collected studies had ≤10 patients, and the remaining studies (11/20, 55%) had >10 patients. The average sample size was 6.2 (SD 2.56) patients in the former studies and 116.5 (SD 111.7) patients in the latter. The average sample size in studies on robotics-based systems was 5.8 (SD 2.5) patients, studies on telemedicine-based systems was 67.5 (SD 61.5) patients, and studies on mHealth systems was 90.3 (SD 112.4) patients. This significant difference in patient sample size can be attributed to the system type. Clearly, mHealth-based systems are more accessible than both robotics-based system and telemedicine-based systems to the patients. This is important to highlight because accessibility is essential to the successful self-management of patient health, which is the goal of AI-based CHI systems.

### Functions and Type of AI Models in CHI Systems

#### Overview

The development of AI has enabled personalized health care [64] at all phases of patient care and treatment. Therefore, it is anticipated that this will assist in addressing the most challenging problems facing precision medicine [65]. This was realized through ML, which yields a predictive model through patient data. For example, an ML-based system can calculate and provide personalized insulin injection amounts for patients with diabetes [15] or supply personalized smoking cessation messages [53]. Prediction also enables classification and detection. The studies in this review present systems that predict exacerbations [45], arrhythmia [58], retina screening [60], or speech detection [47]. These systems are examples of the implementation of AI for classification and detection. However, AI is not limited to ML. Some studies have implemented AI for computer vision, particularly in the field of robotics, to support the rehabilitation of patients with dementia [41,43].

In addition to providing personalized health care, AI enables big data to be efficiently shared across all health care consumers who are the key stakeholders in patient-centered and patient-driven care [66]. Information sharing exposes patients to more flexible options, including prompt and affordable medical assistance [67], allowing them to become more independent and decentralized from the conventional medical structure [68]. For example, this was demonstrated in a study on the use of an AI-based insulin therapy mobile phone app, in which the authors highlighted the ability to store and use large amounts of previous glucose results on a mobile phone system [42]. Furthermore, another study reported that patient data were continuously and passively recorded, and patients were called in for in-person clinic visits as needed [50]. As a result, clinicians and patients could save time [50,51], which is a benefit provided by many patient monitoring and early diagnosis systems. One study reported that 99% of the study participants agreed that the AI-based CHI system could save patient time [60].

#### Algorithms

The AI algorithms used in the selected studies are presented in Table 2. Deep learning (3/20, 15%) and Bayesian networks (3/20, 15%) were the most widely used algorithms. It should be noted that all 3 studies that implemented deep learning [56,59,60] were conducted recently, although this is reported to be due to recent breakthroughs in deep learning and its exceptional performance [69]. Furthermore, deep learning is known to have numerous applications across diverse areas, including health care [70]. That is, deep learning was used for surveillance in general health management [56,59] and decision support in chronic condition–related systems [60]. Nonetheless, deep learning in these systems aims to accurately predict and assist patients with self-health management.

In addition, Bayesian network algorithms were used as much as deep learning in the reviewed studies. In the field of medicine, where gathering data may be costly, difficult, or even impossible [71], this advantage is more significant. Studies that used Bayesian networks focused on general health management [48] and chronic health management [45,51]. However, these studies were consistent in that they aimed to use patients’ previous behaviors or conditions to provide personalized suggestions. The limitation of patient data impedes the use of this algorithm.

Other algorithms used include nearest neighbors [43], k-nearest neighbors [46], artificial neural networks [42], ensemble methods [48], support vector machine [49], Gaussian mixture models [49], and partition around medoids [46].

#### Performance

Some studies also reported the performance of the AI algorithms, as shown in Table 2. The performance of the AI algorithms was evaluated based on accuracy or area under the receiver operating characteristic curve in the reviewed studies. Overall, 6 (30%) out of 20 studies indicated the accuracy of the AI system, of which 5 (83%) [45-47,58,60] reported accuracy greater than 90% or an area under the receiver operating characteristic curve of 0.9. In addition to these systems, OnTrack, a system for the prediction and prevention of dietary lapses, exhibited an accuracy of 72% [48]. The detection accuracy of retina screening [60], arrhythmia [22], and exacerbation was reported to be 96.5%, 97.5%, and 93% (Bayesian network model) [45], respectively. One study reported an average accuracy of 90% for correctly generated therapy plans [46], and another study reported an average accuracy of 97.69% for a voice-based mobile calorie calculation system [47]. In general, the reported AI-based CHI systems were highly accurate. However, an average accuracy difference was observed depending on the algorithm, 97% for deep learning and 82.5% for Bayesian network models, which could be inferred from the previously explained difference in the AI algorithm mechanism.

## Discussion

### Principal Findings

This is the first scoping review focusing on AI-based CHI systems and reporting the outcomes and perceptions related to these systems. The findings show the trend in AI-based CHI systems and provide essential insights into the direction in which this field is proceeding. This review found that various AI-based CHI systems with different functions assist with managing multiple diseases, including both chronic and nonchronic conditions. The geographic locations of the publications were spread among North America (9/20, 45%), Europe (8/20, 40%), Asia (2/20, 10%), and South America (1/20, 5%). However, compared with the number of such products or applications on the market, studies validating their usability and reporting on patient outcomes are limited. Furthermore, many studies included a small sample size of patients and were targeted at collecting patient perceptions toward using the systems; thus, the conclusions tend more toward preliminary feedback for improvements than toward formal validation of patient outcomes.

Most of the studies identified focused on mHealth apps. This is potentially because we were specifically interested in patient-facing personalized systems. However, future studies need to investigate the adoption and sustainable use of the systems, which has been found to be a major issue in mHealth and telehealth in general [72,73]. In addition, the AI method or algorithm used most was supervised learning models. However, it is noteworthy that the interpretability issue of ML has drawn wide attention, and discussions are still ongoing regarding whether a black box ML model should be used in health care, especially for high-stake applications [74].

For the 20 ML-based systems investigated, only 5 (25%) reported their accuracy. The accuracy of those systems’ reports is typically based on the original training. It is not clear how generalizable accuracy is when the system is used on a daily basis for unknown purposes. Maintenance and updating of the system to reflect latest changes in clinical guidelines or databases may also affect its accuracy in the future and have been highlighted in the clinical decision support system field [75]. Furthermore, most of the studies (17/20, 85%) showed somewhat positive patient perception, demonstrating the potential of AI-based CHI system to benefit patients. However, such a review may have a *self-selection effect*—negative findings may not have been published. Future studies could focus on the difficulties encountered, challenges identified, and lessons learned to realize the benefits of such systems.

### Implications and Future Work

The consumer informatics systems are on their way to become a fundamental part of entire health care system, especially after the recent pandemic. The use of telehealth and various mHealth technologies has increased significantly since the beginning of the pandemic [76]. There is also a lot of emphasis on establishing personalized care and home and patient-centered medical care, and AI-supported stronger CHI systems might help fulfill this goal in the overall health care industry. This review also showed that designing user-friendly AI-based CHI solutions to improve patient engagement is critical. It is also critical to create systems that can be integrated with and send data to clinical informatics systems (such as electronic health records) to have a more collaborative nature of work for better decision-making about the patients’ overall health. Future studies should explore how these systems function in three scenarios: (1) when adopted by underserved populations with increased use of smart technologies (smartphones, etc), (2) when used by patients with chronic conditions to manage their care, and (3) when used for preventive care.

### Limitations

One of the limitations of this study is the use of only 1 database; PubMed is the largest database in the medical domain, so it would be sufficient for a scoping review. In addition, our study only focused on the patient-facing systems and patient-reported outcomes. Future studies should also focus on other stakeholders’ perceptions [77]. For example, clinician perceptions on such systems will be important to understand, especially when clinician instruction, prescription, or recommendation is needed to encourage adoption [78].

### Conclusions

Our review identified AI-based CHI systems for a wide range of diseases and purposes. The review also shows that this area has been understudied compared with clinical AI systems in the past years; however, it has gained some momentum since 2020 and will potentially be studied more, given the increased attention toward and encouragement for technology-based personalized care and patient-centered care in the health industry. Studies found favorable patient perceptions in general, which showed potential positive acceptance among patients, although the sample size of several studies was small. Future research should look for evidence of inherited challenges, including the black box issue of ML and sustainable use of mHealth and telehealth, and possible solutions that have been identified in related fields.

## Appendix

Multimedia Appendix 1 PRISMA (Preferred Reporting Items for Systematic Reviews and Meta-Analyses) guidelines.

## Abbreviations

AI: artificial intelligence
CHI: consumer health informatics
MeSH: Medical Subject Headings
mHealth: mobile health
ML: machine learning
PRISMA-ScR: Preferred Reporting Items for Systematic Reviews and Meta-Analyses extension for Scoping Reviews
